# Elemental bioimaging and transcriptomics reveal unchanged gene expression in mouse cerebellum following a single injection of Gadolinium-based contrast agents

**DOI:** 10.1038/s41598-023-33066-6

**Published:** 2023-04-26

**Authors:** Henning Richter, Anke Koke, Patrick N. Soschinski, Louise F. Martin, Patrick Bücker, Michael Sperling, Uwe Karst, Alexander Radbruch, Anika Witten, Astrid Jeibmann

**Affiliations:** 1 Diagnostic Imaging Research Unit, (DIRU), Clinic for Diagnostic Imaging, Department of Clinical Diagnostics and Services, Vetsuisse Faculty, University of Zurich, Winterthurerstraße 258C, 8057 Zurich, Switzerland; 2 Institute of Neuropathology, University Hospital Münster, Pottkamp 2, 48149 Münster, Germany; 3 Institute of Laboratory Animal Science, Vetsuisse Faculty, University of Zurich, Winterthurerstraße 260, 8057 Zurich, Switzerland; 4 Institute of Inorganic and Analytical Chemistry, University of Münster, Corrensstraße 30, 48149 Münster, Germany; 5 Clinic of Neuroradiology, University Hospital Bonn, Venusberg Campus 1, 53127 Bonn, Germany; 6 Core Facility Genomics, Medical Faculty, University of Münster, Domagkstrasse 3, 48149 Münster, Germany

**Keywords:** Experimental models of disease, Gene expression

## Abstract

Gadolinium (Gd) deposition in the brain, first and foremost in the dentate nucleus in the cerebellum, following contrast enhanced MRI, rose awareness of potential adverse effects of gadolinium-based contrast agent (GBCA) administration. According to previous in vitro experiments, a conceivable side-effect of Gd deposition could be an alteration of gene expression. In the current study, we aimed to investigate the influence of GBCA administration on gene expression in the cerebellum of mice using a combination of elemental bioimaging and transcriptomics. In this prospective animal study, three groups of eight mice each were intravenously injected with either linear GBCA gadodiamide, macrocyclic GBCA gadoterate (1 mmol GBCA/kg body weight) or saline (NaCl 0.9%). Animals were euthanized four weeks after injection. Subsequently, Gd quantification via laser ablation-ICP-MS and whole genome gene expression analysis of the cerebellum were performed. Four weeks after single application of GBCAs to 24–31 days old female mice, traces of Gd were detectable in the cerebellum for both, the linear and macrocyclic group. Subsequent transcriptome analysis by RNA sequencing using principal component analysis did not reveal treatment-related clustering. Also differential expression analysis did not reveal any significantly differentially expressed genes between treatments.

## Introduction

Gadolinium (Gd), a heavy metal of the lanthanide group, has been used as a base for contrast agents in MRI for the last three decades. As free ion, it is able to inhibit calcium channels through competitive binding. To overcome cellular toxicity, chelated forms of Gd, classified as linear or macrocyclic (ionic or non-ionic), have been manufactured. In general, macrocyclic Gadolinium-based contrast agents (GBCAs) are thermodynamically and kinetically more stable than linear GBCAs^1,2^.

Deposition of Gd in the human brain has been reported after GBCA application^3–5^. In 2014, Kanda et al.^3^ showed increased signal intensities in the human dentate nucleus and globus pallidus after repetitive application of linear GBCAs. Their results were confirmed by large numbers of retrospective studies in both humans and animals^4^ but no adverse effects were observed. This may partly be due to the fact that potential adverse effects of GBCAs are masked as they are used in the clinical context for patients already showing neurological symptoms or lack of information about GBCA application may have prevented correct assignment to related symptoms^6^.

Although clinical consequences of Gd retention in the brain were still unclear, in 2017 the European Medicines Agency (EMA) embraced a precautional position in patients safeguard, and suspended the marketing authorization of linear GBCAs in the EU^7^, with the exception of few linear GBCAs for special applications. However, investigations on this topic are still of broad interest, as linear GBCAs are further used outside the EU, and macrocyclic GBCAs continue to be applied worldwide.

A large number of studies have been performed in animals to assess the occurrence of Gd deposition: Gd deposition was mainly reported with linear agents^8–10^, while deposition associated with macrocyclic agents remains more debated^11,12^. Until now no histological alterations within the brain were discovered in animal studies^13,14^, or in a human autopsy study^15^. Only one study describes a significant effect of linear GBCAs on small fibers in the footpads of mice^16^. However, this still does not exclude the possibility that more subtle tissue alterations were missed by histological techniques as, for example, previous studies found gene expression alterations in cultured macrophages^17^ and hepatocytes^18^ or metabolic changes in dopaminergic neurons after GBCA exposure^19^, although in one study after serial injections of GBCAs no general pathway deregulation was found in rat brains^6^. In order to detect changes to gene expression and biological pathways potentially altered by a single GBCA administration, we examined the whole transcriptome by RNA sequencing of the mouse cerebellum after application of linear and macrocyclic GBCAs gadodiamide and gadoterate.

## Results

### Gene expression analysis

After quality control, alignment and normalization of the RNA sequencing data (Supplemental Figs. 1, 2, 3), an unsupervised sample clustering was performed using principal component analysis (PCA)^20^. If gene expression profiles of samples are similar, they will cluster closer together in a PCA plot. For our data set the PCA plot does not show treatment related clustering (Fig. 2A).

**Figure 1:**
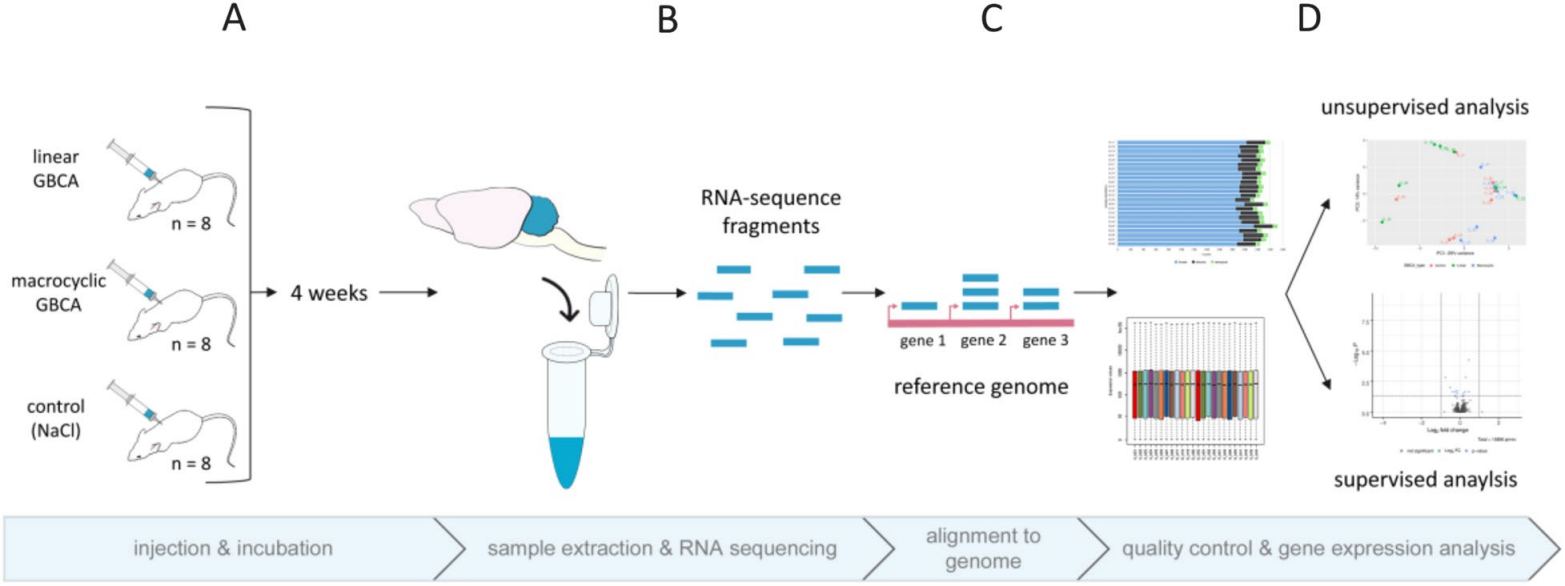
Study design of gene expression analysis. Three groups of mice were injected with either linear GBCA, macrocyclic GBCA or saline (**A**). After four weeks, mice were sacrificed and RNA from whole cerebella was extracted and sequenced (**B**). The resulting RNA fragments were aligned to the mouse reference genome (**C**). Quality controls of RNA sequences were performed to ensure unbiased analysis of high-quality data. Gene expression analysis was performed in unsupervised and supervised form (**D**). Unsupervised analysis by principal component analysis (PCA) was conducted to compare the whole gene expression between samples. In the supervised analysis gene expression profiles were compared between treatment groups to identify up- or downregulated genes.

**Figure 2:**
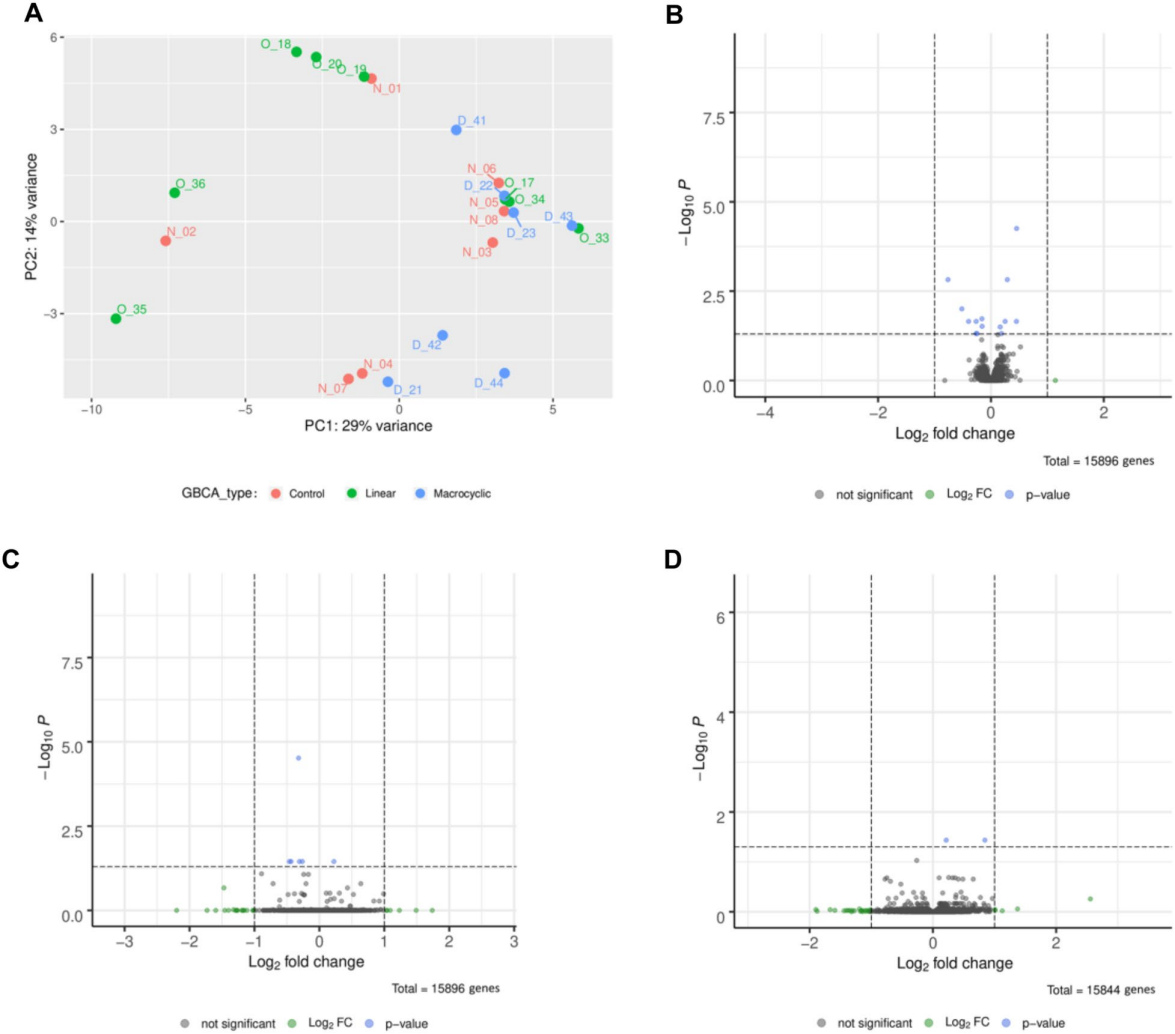
Unsupervised and supervised analysis of gene expression profiles. Principal component analysis of gene expression profiles (**A**) O = linear GBCA; D = macrocyclic GBCA; N = control treatment. Volcano plots (**B**–**D**) of all reference mouse genes differentially expressed between linear GBCA and control treatment (**B**), macrocyclic GBCA and control treatment (**C**) and linear GBCA and macrocyclic GBCA treatment (**D**). Total = number of genes identified in all 16 samples of a comparison. *p*-value (blue) = Gene with significant *p*-value; Log2FC (green) = Gene with |L2FC|≥ 1; not significant (grey) = Gene with insignificant *p*-value and |L2FC|< 1.

**Figure 3:**
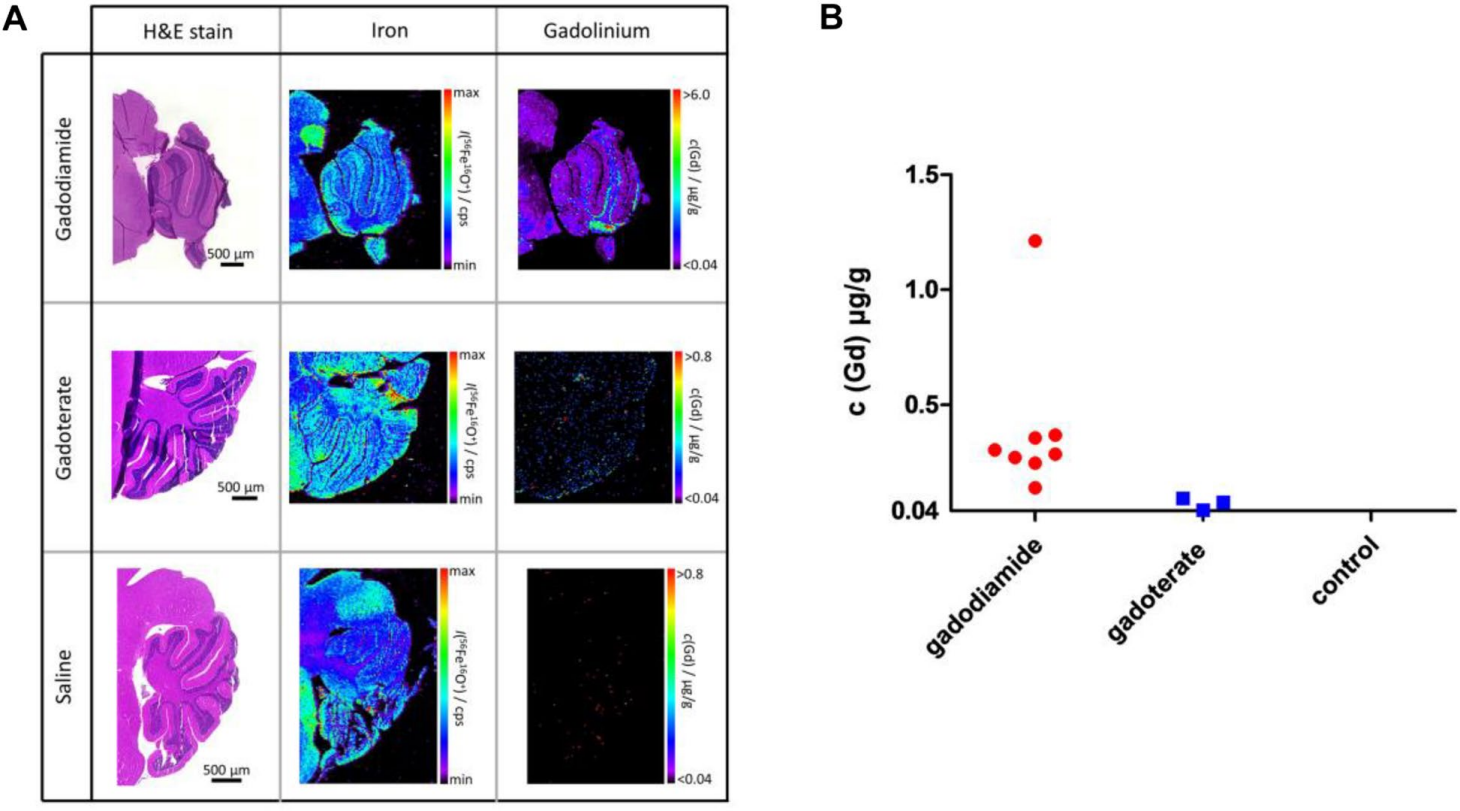
Distribution of gadolinium and iron in the cerebellum of mice after GBCA or control treatment. Example photomicrographs of H&E-stained cerebella after gadodiamide, gadoterate and saline administration with respective qualitative iron and quantitative gadolinium distribution analyzed by LA-ICP-MS (**A**). Gd concentration in each sample is illustrated (**B**), if above the limit of quantification of 0.040 µg/g (gadodiamide group: 8/8 samples above limit of quantification; gadoterate group: 3/8; control group: 0/8).

Differences in gene expression between treatments are visualized using volcano plots (Fig. 2). Genes that are downregulated after GBCA treatment have a negative log twofold change (L2FC), while upregulated genes have a positive L2FC (plotted on the x-axis). Significantly differentially expressed genes were defined as having an adjusted *p*-value ≤ 0.05 (plotted on the y-axis) and a |L2FC|≥ 1 and are shown in red (Fig. 2). Genes which only fulfill one condition are shown in green (|L2FC|≥ 1) or blue (adjusted *p*-value ≤ 0.05). The numbers of genes identified in all 16 samples of a comparison with any *p*-value and any L2FC are given at the bottom of the plots (‘Total’).

15,896 genes were identified comparing control vs. linear GBCA treatment, 15,896 genes comparing control vs. macrocyclic GBCA treatment, 15,844 genes comparing linear GBCA vs. macrocyclic GBCA treatment. No genes fulfilled the criteria of significance (|L2FC|≥ 1 and adjusted *p*-value ≤ 0.05) for any treatment comparison.

Thus, in the current study, no changes of gene expression in transcriptomes could be identified between the groups investigated.

### Gadolinium quantification

Gd concentration in the cerebellum was determined using LA-ICP-MS and results are presented in Fig. 3. For all investigated samples, HE-stained images provide an overview of the cerebellum (Fig. 3A). Additionally, qualitative iron distribution is depicted for structural reference. In comparison to the respective Gd distributions, a spatial correlation between iron and Gd was observed. Gd deposition was especially found in the deep cerebellar nuclei (DCN) of the mice. Gadodiamide application resulted in the highest Gd concentration (0.40 µg/g ± 0.34 µg/g) which differed from concentrations determined after injection of gadoterate (5 out of 8 samples show Gd concentrations < 0.040 µg/g and therefore < LOQ, limit of quantification) and the control group (8 out of 8 samples show Gd concentrations < LOQ) (Fig. 3B). Statistics could not be performed as the majority of values was < LOQ.

## Discussion

Taken together, gene expression profiles reflect that there is no deregulation of biological pathways induced by application of GBCAs, even though Gd was detectable. These results are in line with another recent study investigating gene expression analysis of deep cerebellar nuclei in rats at a later time point (11 weeks) after administration of high and repetitive doses of gadodiamide and gadobutrol^6^. Similar to our data, they could not identify a general pathway deregulation in the deep cerebellar nuclei of treated animals. Additionally, we provide quantitative information about Gd in the cerebellum concurrently to gene expression analysis.

LA-ICP-MS proved to be a suitable tool not only to quantify the Gd deposition in the tissue but also to provide information about the spatial distribution of the elements. While the concentration sensitivity of a highly spatially resolved LA-ICP-MS analysis is typically below the one of a classical ICP-MS analysis with liquid introduction setup due to a limited mass flow into the ICP-MS (e.g., a volume element of 10 µm × 10 µm × 10 µm corresponds to a total volume of only 1 pL), the provided spatial resolution strongly facilitated the selectivity of the analysis, as the structure of interest could be precisely defined through the image^21^. With this, traces of Gd were detected and quantified by LA-ICP-MS in the cerebellum of mice that were treated with linear and macrocyclic GBCAs, with the amount being higher after linear GBCA and very low after macrocyclic GBCA administration. Thus, the results are in line with the generally accepted concept that the less stable linear GBCAs cause significant Gd deposition while the kinetically more stable macrocyclic GBCAs are retained to a lesser extent. Predominant deposition of Gd in the DCN of mouse cerebella and spatial correlation with iron is comparable to findings in humans^15^.

This study was conducted as add-on to another study performed for teaching purposes. This meets the 3R requirements (reduce, refine, replace) and helps to reduce the number of experimental animals. Although studies were unrelated, without interference and completely independent from each other, the time point of animal sacrifice was predetermined. However, previous studies showed that the majority of macrocyclic contrast agents are washed out after four weeks in rodents^22^. Thus, it is possible that an acute cellular response to GBCA treatment took place before sacrifice, and that response had become undetectable four weeks after injection. However, as there was no major alteration of gene expression profiles after 4 weeks in our study, we can also conclude that no chronic changes were induced by GBCAs even if traces of them were still detectable in the cerebellum.

Differentially expressed genes such as IL-6, IL-1ß or genes of the NF-kappaB pathway that have been reported in the in vitro studies after GBCA treatments^17,22,23^ could not be verified in the current study. Taken together, no changes of gene expression profiles in the cerebellum may reflect the fact that no neurological cerebellar symptoms could be ascribed to Gd presence in the brain.

However, it has to be taken into account, that for gene expression analysis, whole cerebella were investigated, as extraction of the deep cerebellar nuclei of mice is technically challenging. Gd deposition is found mainly in the deep cerebellar nuclei of rodents and large animals, as well as in the dentate nucleus of humans^9,15,22^. Presence of other cerebellar structures in our RNA extraction may dilute the gene pool and mask the identification of significantly differentially expressed genes caused by GBCA administration. Regrettably, the relative percentage of weight that the DCN represents in relation to the entirety of the cerebellum remains undetermined and cannot be subject to further evaluation.

## Conclusion

We applied the combination of elemental bioimaging and transcriptomics to show that the administration of linear GBCAs results in higher Gd levels in mouse cerebella than the administration of macrocyclic GBCAs and despite traces of Gd found in the cerebellum, no genes were significantly differentially expressed for none of the GBCA classes. We conclude that four weeks after single application of GBCAs, no gene expression alterations in the cerebellum are detected.

## Methods

### Mouse study

This animal study was conducted as “add-on” to a study for teaching purposes. 24 female mice (RjOrl:SWISS) were included (24–31 days after delivery from Janvier-Labs, Le Genest-Saint-Isle, France) and randomly subdivided into 3 groups with 8 animals, injected with linear GBCA gadodiamide (Omniscan®, GE Healthcare AG, Wädenswil, Switzerland), macrocyclic gadoterate meglumine (Dotarem®, Guerbet AG, Paris, France), or saline (NaCl 0.9%) (Fig. 1 A). Injections of GBCAs were performed once at a dose of 1 mmol/kg BW, into the tail vein. Controls received injections of NaCl (2 ml/kg, i.v.). Injections were performed on awake and healthy animals positioned within a restrainer. This study was performed in accordance with the Swiss Animal Welfare Act (TSchG, 2005) and Swiss Animal Welfare Ordinance (TSchV, 2008). The study was approved by the Swiss Federal Veterinary Office Zurich (animal license number ZH029/19) and performed by two veterinarians as add-on to another animal experiment (ZH214/16 +) with a focus on teaching purposes. The combination of the studies in accordance with 3R principles allowed a reduction of experimental animals. The study is in compliance with the ARRIVE guidelines.

### Sacrifice, tissue harvesting and sample preparation

Animals were euthanized by carbon dioxide inhalation according to the American Veterinary Medical Association guidelines^24^, 4 weeks after injection (Fig. 1B). Death of the animals was confirmed based on the reversal of the heartbeat and respiration. Right cerebellar hemisphere was stored at −80 °C. Left hemisphere was formalin fixed, paraffin-embedded and hematoxylin–eosin stained.

### RNA-Sequencing

RNA was extracted using the Direct-zol-96 RNA kit (Zymo Research, Irvine, CA, USA) according to standard protocols. Quality control was conducted using the RNA ScreenTape (Agilent 2200 TapeStation, Agilent, St. Clara, CA, USA) yielding RNA integrity number (RIN) values of seven and higher. The mRNA was enriched using NEBNext Poly(A) mRNA Magnetic Isolation Module (New England Biolabs, Ipswich, MA, USA). For the strand-specific cDNA NGS library preparation, the NEBNext®Ultra II Directional RNA Library Prep Kit for Illumina (NEB #E7760S/L, , New England Biolabs, Ipswich, MA, USA) was used according to manufacturer’s instructions. Library size was controlled and quantified by D1000 ScreenTape (Agilent, St. Clara, CA, USA) and the NEBNext Library Quant Kit for Illumina (New England Biolabs, Ipswich, MA, USA). The final cDNA library sequencing took place on an Illumina NextSeq 500 System (Illumina, San Diego, CA, USA) with 75 single end reads (Fig. 1C).

### Gene expression analysis

The sequence information was stored in the FASTQ format. The quality of the obtained sequences (reads) was evaluated using FASTQC 0.11.5^25^. Reads were aligned with STAR 2.7.3a^26^ to the mouse GRCm38 release 99 reference genome including 55,476 known genes (Fig. 1D). Alignment quality control was performed using QUALIMAP (v.2.2.2-dev) BAMQC and RNASeq QC^27^ (Fig. 1D). A gene count matrix was generated using HTSeq count 0.12.4 ^28^ with default parameters for single-end reads and gene wise counting. NOISeq 2.30.0 ^29,30^ was used to assess the quality of the count matrices. Normalization of read counts and differential expression analysis was performed by DESeq2 1.28.0^31^ (Fig. 1D). The read counts were fit to a model which compared the treatments. A read-count filtering as suggested by DESeq2 manual was implemented: Genes with a read count ≤ 10 in ≤ 8 samples were excluded. Using Benjamini–Hochberg correction^32^ *p*-values were adjusted for multiple testing. Genes with an adjusted *p*-value < 0.05 and a log twofold change (|L2FC|) above 1 in expression were marked as differentially expressed.

### Sample and standard preparation for LA-ICP-MS

Paraffin-embedded specimens of mouse cerebellum were cut into 10-µm sections (Leica CM3050S, Leica Biosystems Nussloch GmbH, Nussloch, Germany). To quantify the Gd concentration in cerebellar tissue, eight matrix-matched gelatine standards were prepared in a concentration range from 0 to 10 µg/g according to previously established protocols. The gelatine standards were spiked with a solution of GdCl_3_∙ 6 H_2_O (Alfa Aesar, Haverhill, MA, USA) to obtain the desired Gd concentration and homogenized at 50 °C, cooled, cut and mounted onto a slide. An inductively coupled plasma-mass spectrometry (ICP-MS) instrument model iCAP TQ (Thermo Fisher Scientific, Bremen, Germany) was used to confirm the Gd concentration and homogeneity of the gelatine standards by means of total digestion.

### LA-ICP-MS analysis

For spatially resolved analysis, the hyphenation of an imageBIO266 laser ablation system (Elemental Scientific Lasers, Bozeman, MT, USA) and the iCAP TQ ICP-MS was used. For analysis, a spot size of 25 µm and a stage speed of 100 µm/s were chosen. For calibration, 11 lines of each standard were ablated, with the first line being excluded of evaluation, as it could have a slightly different width due to laser warmup and not being next to a previously ablated line. Evaluation was performed utilizing the software ImaJar (3.64b, by Robin Schmid). Limit of quantification (LOQ) was defined as ten times the standard deviation of the blank divided by the calibration slope. For this setup, an LOQ of 0.040 µg/g was determined. Calculated absolute blank concentrations were below the LOQ threshold. Calibration curves showed r^2^ > 0.999 throughout all runs. Statistics could not be performed as the majority of Gd concentration values was < LOQ. The described LA-ICP-MS setup was used to analyze the qualitative iron and quantitative gadolinium distributions.

## Supplementary Information

Supplementary Information.

## Data Availability

The datasets generated and analysed during the current study are available in the Zenodo repository, under the following link: https://doi.org/10.5281/zenodo.7413768.

## Abbreviations

Gd: Gadolinium
MRI: Magnetic resonance imaging
GBCA: Gadolinium-based contrast agent
PCA: Principal component analysis
EMA: European Medicines Agency
RIN: RNA integrity number
FASTQ format: Text-based format for storing both a biological sequence (usually nucleotide sequence) and its corresponding quality scores
L2FC: Log twofold change
LOQ: Limit of quantification
ICP-MS: Inductively coupled plasma-mass spectrometry
LA: Laser ablation
3R: Reduce, refine, replace
