# Supplementary material for: Elemental bioimaging and transcriptomics reveal unchanged gene expression in mouse cerebellum following a single injection of Gadolinium-based contrast agents
Source: Sci Rep. 2023 Apr 26;13:6844. doi: 10.1038/s41598-023-33066-6 (PMC10133442; doi:10.1038/s41598-023-33066-6)
Supplement: Supplementary file 1 — Supplementary Information. [file 41598_2023_33066_MOESM1_ESM.pdf]

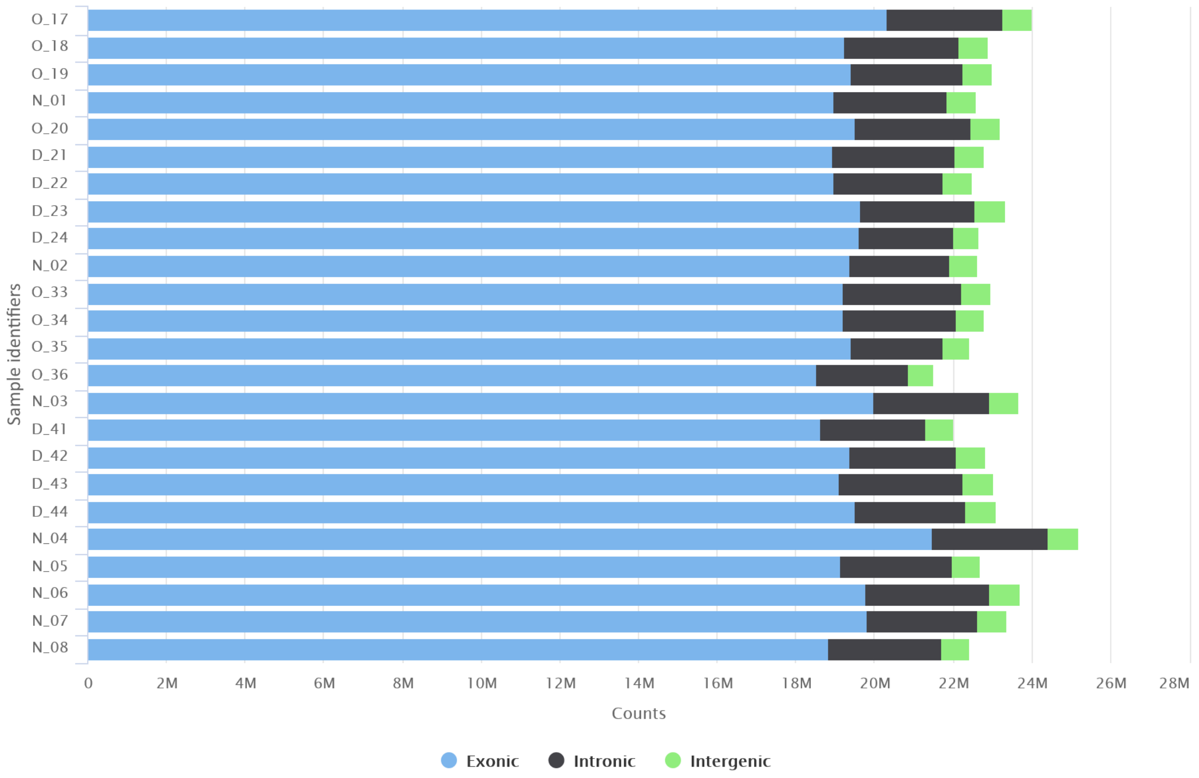

Supplemental figure 1: Genomic origin of counts per sample

Visualization of the abundance of RNA sequencing products (reads) originating from (exonic, blue) and non-coding (intronic: black and intergenic: green). Introns are mRNA components that are removed in a process called splicing. Exons contain the actual genetic information that is translated into an amino acid sequence. Intergenic regions are located between two genes. Total abundance (counts) is given in million (M). Sample identifiers on the left indicate the GBCA treatment and animal number. M = million counts; O = linear GBCA (n=8), D = macrocyclic GBCA (n=8), N = control (n=8).

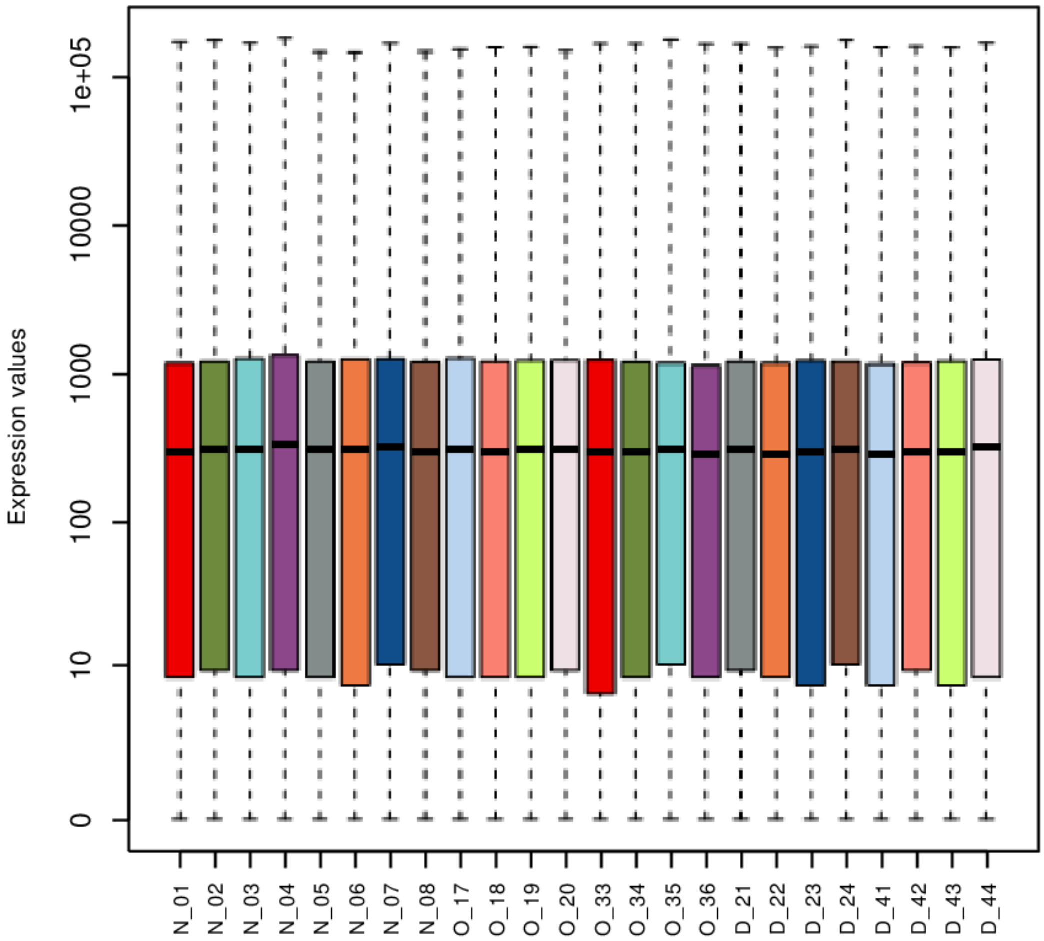

Supplemental figure 2: Expression values of protein coding transcripts per sample

Boxplots indicate the total number of protein coding RNA sequencing products (reads) per sample identifier. Boxes represent quartiles, the black horizontal line indicates the median, whiskers show minima and maxima. Sample identifiers indicate the GBCA treatment and animal number. O = linear GBCA (n=8), D = macrocyclic GBCA (n=8), N = control (n=8). A lower or higher expression value in one sample could indicate differences in sequencing quality between samples.

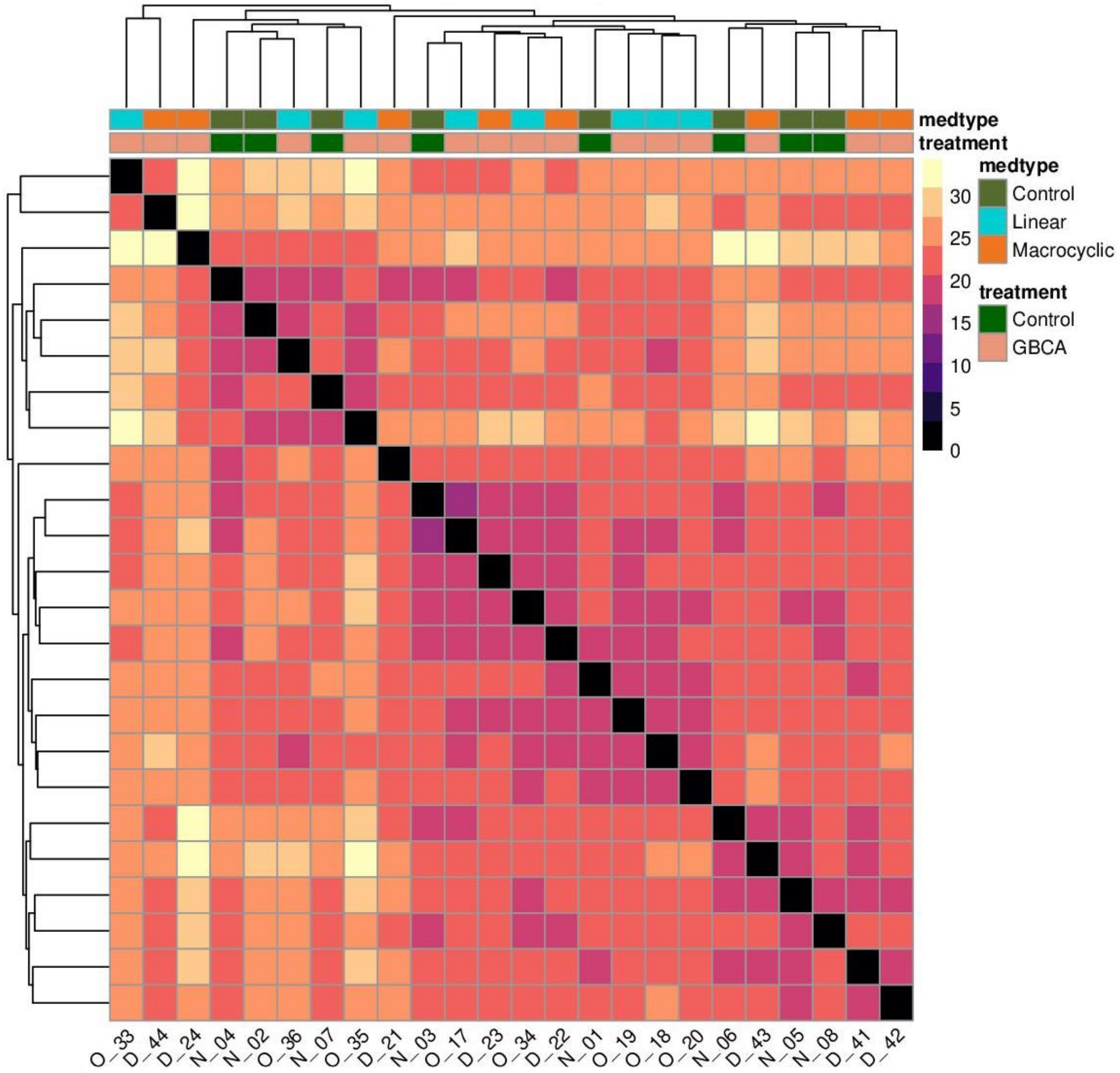

Supplemental figure 3: Sample-to-sample heatmap of gene expression profiles.

Samples are clustered to each other based on their gene expression profiles. The dendrogram on the left and top indicates hierarchical relationships between the samples and thereby helps to allocate samples to clusters. The “average” algorithm was used to perform the hierarchical clustering. Clustering distance: Euclidian. O = linear GBCA (n=8), D = macrocyclic GBCA (n=8), N = control (n=8).
